# Iron Overload-Associated Oxidative Stress and Immune Cell Dysfunction in Thalassemia: Integrative Analysis of Hematological, Biochemical, and Flow Cytometric Biomarkers

**DOI:** 10.3390/antiox15040482

**Published:** 2026-04-14

**Authors:** Sirichai Srichairatanakool, Narisara Paradee, Bishant Pokharel, Yanping Zhong, Onsaya Kerdto, Wuttipat Kiratipaisarl, Adisak Tantiworawit, Chatree Chai-Adisaksopha, Somdet Srichairatanakool

**Affiliations:** 1 Division of Hematology, Department of Internal Medicine, Faculty of Medicine, Chiang Mai University, Chiang Mai 50200, Thailand; sirichai.srichai@cmu.ac.th (S.S.); adisak.tan@cmu.ac.th (A.T.); chatree.chai@cmu.ac.th (C.C.-A.); 2 Department of Biochemistry, Faculty of Medicine, Chiang Mai University, Chiang Mai 50200, Thailand; narisara.p@cmu.ac.th (N.P.); bishant_p@cmu.ac.th (B.P.); yanping_z@cmu.ac.th (Y.Z.); onsaya35@gmail.com (O.K.); 3 School of Medical Technology and Artificial Intelligence, Youjiang Medical University for Nationalities, Baise 533000, China; 4 Department of Community Medicine, Faculty of Medicine, Chiang Mai University, Chiang Mai 50200, Thailand; wuttipat.k@cmu.ac.th

**Keywords:** thalassemia, iron overload, oxidative stress, reactive oxygen species, redox-active iron, granulocytes, antioxidant defense

## Abstract

Thalassemia is a hereditary hemoglobinopathy characterized by ineffective erythropoiesis, chronic hemolysis, and transfusion-related iron overload, which collectively contribute to oxidative stress and organ dysfunction. The present study aimed to investigate the relationships between iron metabolism, oxidative stress biomarkers, and immune cell function across different clinical conditions. Peripheral blood samples were obtained from healthy individuals and patients with iron deficiency anemia, obesity, thalassemia trait (TT), β-thalassemia HbE (BTE), and β-thalassemia major (BTM). Hematological parameters were measured using automated hematology analyzers, while biochemical indicators, including liver enzymes and bilirubin, were determined using clinical chemistry assays. Iron overload was evaluated using serum iron parameters and T2*-weighted magnetic resonance imaging. Oxidative stress biomarkers, including reduced glutathione, thiobarbituric acid-reactive substances, and total antioxidant capacity, were assessed spectrophotometrically. Flow cytometric analysis was used to measure reactive oxygen species, redox-active iron, and lipid peroxide levels in granulocytes and lymphocytes. Thalassemia patients exhibited severe anemia, elevated liver enzymes, increased bilirubin levels, and significant alterations in iron metabolism compared with healthy controls. Hepatic iron accumulation was more common than cardiac iron deposition, particularly in BTE patients. Granulocyte oxidative burst activity was significantly reduced in thalassemia patients, whereas lymphocyte responses remained relatively preserved. Increased variability in glutathione levels suggested activation of intracellular antioxidant defense mechanisms in response to chronic oxidative stress. These findings highlight the complex interplay between iron overload, oxidative stress, and the immune cell dysfunction associated with thalassemia, thereby providing insights into improved monitoring and therapeutic strategies.

## 1. Introduction

Thalassemia represents one of the most common inherited hemoglobin (Hb) disorders worldwide and is characterized by the defective synthesis of globin chains that leads to ineffective erythropoiesis and chronic hemolytic anemia [1,2]. The β-thalassemia syndromes included in this study are categorized as BTM, β-thalassemia intermedia (TI), BTE, and TT, all of which are particularly prevalent in Southeast Asia and other regions associated with a high carrier frequency [2,3]. Patients with severe forms of the disease often require lifelong blood transfusion therapy to maintain adequate Hb levels; however, repeated transfusions contribute to progressive iron overload and the development of multiple organ complications [4,5]. Hemoglobin E (HbE) is characterized by a point mutation in the β-globin gene at codon 26 (Gln→Lys), resulting in structurally abnormal Hb and a mild β-thalassemia phenotype when co-inherited with β-thalassemia mutations [6,7].

Iron overload is a major clinical concern in thalassemia and results from both repeated blood transfusions and the increased intestinal iron absorption associated with ineffective erythropoiesis [5,8]. Excess iron accumulates primarily in the liver, heart, and endocrine organs, where it promotes the generation of reactive oxygen species (ROS) through redox cycling reactions. These processes contribute to oxidative stress, cellular damage, and progressive organ dysfunction [9]. Magnetic resonance imaging using T2*-weighted techniques (T2*-MRI) has become a reliable non-invasive method for evaluating myocardial and hepatic iron deposition in patients with transfusion-dependent thalassemia [10,11]. In addition to systemic iron overload, increasing evidence indicates that oxidative stress plays a central role in the pathophysiology of thalassemia. Iron-mediated ROS generation, chronic hemolysis, and ineffective erythropoiesis contribute to oxidative damage in erythrocytes and other tissues [9]. Oxidative stress has been associated with lipid peroxidation, depletion of antioxidant defenses, and disruption of normal cellular metabolism in thalassemia patients [12,13]. Antioxidant defense systems, including reduced glutathione (GSH) and other cellular redox regulators, play a critical role in maintaining redox balance and protecting cells from oxidative injury [14,15]. Beyond erythrocyte damage, oxidative stress may also affect immune cell function in thalassemia. Neutrophils and other granulocytes rely on oxidative burst mechanisms to generate ROS required for microbial killing. Alterations in intracellular redox balance may therefore impair innate immune responses and contribute to the increased susceptibility to infections reported in some thalassemia patients [16,17]. However, the relationship between iron overload, oxidative stress, and immune cell functional activity remains incompletely understood.

Biomarkers of oxidative stress and antioxidant defense, including GSH, thiobarbituric acid-reactive substances (TBARSs), and total antioxidant capacity (TEAC), provide valuable insight into systemic redox homeostasis. Furthermore, advances in flow cytometric techniques allow for the direct assessment of intracellular oxidative markers, such as ROS, redox-active iron (RAI), and lipid peroxides (LPOs), within specific immune cell populations. Therefore, the present study aimed to investigate the interplay between iron metabolism, oxidative stress, and immune cell function in thalassemia. By integrating hematological analysis, biochemical parameters, T2*-MRI-based iron assessment, oxidative stress biomarkers, and the flow cytometric evaluation of leukocyte oxidative activity, this study provides a comprehensive assessment of redox imbalance and immune dysregulation across multiple clinical groups. These groups included healthy individuals, as well as those with iron deficiency anemia (IDA), obesity, TT, BTE, and BTM. This study investigated the relationships between iron metabolism, oxidative stress, and immune cell function in thalassemia. We assessed hematological parameters, liver function biomarkers, iron overload status, oxidative stress markers, and leukocyte oxidative responses in healthy individuals and patients with IDA, obesity, TT, BTE, and BTM. This integrative approach was intended to provide a comprehensive understanding of the interplay between iron overload, oxidative stress, and immune dysfunction in thalassemia.

## 2. Materials and Methods

### 2.1. Chemicals and Reagents

2,2-Azino-*bis*-(3-methylbenzothiazoline-6-sulfonic acid) (ABTS) (product number A1888, >98% pure), butylated hydroxytoluene (BHT) (product number W218405, ≥99% pure), hydroxyethyl piperazineethanesulfonic acid (HEPES) (product number H3375, 99.5% pure), meta-phosphoric acid (H_3_PO_4_) (product number 239275, concentration 33.5–36.5%), 2-thiobarbituric acid (TBA) (product number T5500, ≥98% pure), 6-hydroxy-2,5,7,8-tetramethylchroman-2-carboxylic acid (Trolox) (product number 238813, 97% pure), and Ficoll Paque Plus solution (product code GE17-1440-02; with endotoxins at <0.12 unit/mL and a density of 1.077 g/mL) were obtained from Sigma-Aldrich Chemical Company, Saint Louis, MO, USA. Potassium persulfate (product number 216224) was purchased from Merck KGaA, Darmstadt, Germany. 2′,7′-Dichlorohydrofluorescein diacetate (DCFH-DA) (product code R252-10), *N*-(4-diphenylphosphinophenyl)-*N*’-(3,6,9,12-tetraoxatridecyl)perylene-3,4,9,10-tetracarboxydiimide (Liperfluo, LF) (product code L248), and FerroOrange (FO) (product code F374) were purchased from Dojindo Laboratories, Kamimashiki-gun, Kumamoto, Japan. Ellman’s reagent containing 5,5′-dithiobis(2-nitrobenzoic acid) (DTNB) for colorimetric GSH assays was obtained from Wuhan Elabscience Biotechnology Company Limited, Wuhan, Hubei, China. Phosphate buffer (PB) (Catalog number 258595000) and phosphate-buffered saline (PBS) (Catalog number AM9625) solution, pH 7.0, were purchased from Thermo Fisher Scientific, Middlesex Counter, MA, USA. BD FACS™ Lysing solution (Catalog number 349202) and azide-free BD FACSFlow sheath fluid buffered solution, pH 7.8–8.2 (Catalog Number 342003—20 L) and comprising 97.8% water (*v*/*v*), were provided by Becton Dickinson Biosciences Company, San Jose, CA, USA. Blood diluent (product No. 8547167), lysing reagent (product No. 8547166), cleaning solution (product No. 8547170), and quality control materials (product No. 628029) were supplied by Beckman Coulter, Inc., Brea, CA, USA. Reagent assay kits for serum iron (SI) (product No. 05168990190), unsaturated iron-binding capacity (UIBC) (product No. 08058776190), ferritin (product No. 11820982122), aspartate aminotransferase (AST) (product number 04467493190), alanine aminotransferase (ALT) (product number 04467388190), and alkaline phosphatase (ALP) (product number 05166888190), and calibrators (product number 10759350190) and quality control materials (product number 09339795190) were supplied by Roche Diagnostics International AG, Rotkreuz, Switzerland. All the commercial assay reagents and kits were used according to the manufacturer’s protocol and instructions.

### 2.2. Ethical Considerations and Approval

This study was conducted in accordance with the ethical principles of the Declaration of Helsinki and complied with all relevant institutional and national regulations governing research involving human participants. Ethical approval was granted by the Research Ethics Committee (REC) of the Faculty of Medicine, Chiang Mai University (Research ID: 0542; Protocol Code: MED-2566-0542, with officical approval issued on 15 March 2024). Prior to participation, all individuals were informed of the purpose of the study, the procedures involved, any potential risks, and the anticipated benefits. Written informed consent was obtained from every participant before any study-related procedures were performed. Participation was voluntary, and participants were informed that they could withdraw from the study at any time without affecting their medical care or access to healthcare services. All participant information was handled with strict confidentiality. Personal identifiers were removed and replaced with coded identification numbers to maintain anonymity. Research data were securely stored in password-protected electronic databases and locked storage areas accessible only to authorized members of the research team. Blood samples were obtained by trained healthcare professionals using sterile and aseptic techniques to minimize the risk of discomfort or infection. The volume of all blood collected remained within clinically acceptable limits and was considered safe for the participants. All biological samples were handled, stored, and disposed of according to institutional biosafety regulations. Throughout the study, particular attention was given to protecting the safety, dignity, and welfare of all participants.

### 2.3. Patient Recruitment

Participants were recruited from patients with a confirmed diagnosis of thalassemia who attended routine follow-up visits at a participating healthcare facility. Multiple subject groups were included to represent different physiological and pathological states of iron metabolism. Healthy individuals served as controls. IDA represented iron deficiency, while obesity represented a metabolic condition associated with oxidative stress. The thalassemia subgroups in the present study were TT, BTE, and BTM, and their diagnosis was determined by Hb typing or genetic testing; the BTE group formed the largest subgroup because of the high prevalence of BTE in the study region. Eligible participants met the following criteria: confirmed diagnosis of thalassemia (BTM, TI, or BTE), determined by Hb analysis or genetic testing; age of 20 years or older; receiving regular clinical care at a participating hospital or clinic; clinically stable at the time of sample collection without evidence of acute illness or complications; and the ability to understand the study procedures and provide written informed consent. Individuals were excluded if any of the following conditions were present: signs of acute infection, fever, or inflammatory disease at the time of blood collection; history of blood transfusion within the previous 3–4 weeks; presence of other hematological disorders such as sickle cell disease or leukemia; severe medical conditions including acute heart failure, renal failure, or liver failure; participation in experimental treatments that could influence hematological or biochemical parameters; or inability or unwillingness to provide informed consent.

### 2.4. Patient Preparation

Before blood collection, the participants received a clear explanation of the study procedures and provided written informed consent. Participant identity was verified using the hospital identification number or medical record. Clinical and demographic information was collected, including age, sex, thalassemia subtype, transfusion history (including the date of the most recent transfusion), current medications, and overall clinical status. Fasting was not required unless specific laboratory analyses indicated otherwise. For the blood collection procedure, participants were placed in a comfortable seated or reclined position to reduce discomfort and lower the risk of dizziness or fainting.

### 2.5. Blood Collection and Sample Processing

Whole blood samples (10 mL) were collected in heparinized tubes for hematological analysis and leucocyte preparation, and serum or plasma fractions were obtained for biochemical and oxidative stress assays. For leucocyte preparation, 100 μL of heparinized whole blood was treated with 1 mL of 1× BD FACS lysing solution containing 15% formaldehyde and 50% diethylene glycol to lyse red blood cells (RBCs), following the manufacturer’s instructions.

### 2.6. Hematological Analysis

Complete blood count (CBC) analysis was performed using an automated hematology analyzer (AC-T diff2, Beckman Coulter, Inc., Brea, CA, USA).

### 2.7. Biochemical Analysis

Liver enzyme activities, including AST, ALT, and ALP, were determined in serum samples using an automated clinical chemistry analyzer (Cobas 8000 modular series, Roche Diagnostics International AG, Rotkreuz, Switzerland) using kinetic photometric methods according to the manufacturer’s protocols [18]. The assays were performed using kinetic photometric methods according to the manufacturer’s protocols. The results of the enzyme activities were expressed in units per liter (U/L).

### 2.8. Iron Overload Assessment

#### 2.8.1. Body Iron

T2*-MRI was specifically applied for iron overload assessment, rather than subtype classification, and used to evaluate myocardial and hepatic iron deposition. Myocardial and hepatic iron deposition were evaluated using the T2*-MRI technique performed using a GE 1.5-T MRI scanner (SIGNA Explorer, GE Healthcare, Milwaukee, WI, USA) equipped with cardiac imaging software (GE Advantage Workstation StarMap version 4.0, GE Healthcare, Milwaukee, WI, USA). During the examination, participants were positioned supine and scanned using a phased-array coil. For hepatic assessments, breath-hold multi-echo gradient-echo images were acquired across the liver parenchyma. T2* relaxation maps were generated using the GE workstation or dedicated post-processing software. For myocardial measurements, regions of interest (ROIs) were placed within the interventricular septum while carefully avoiding the ventricular blood pool and susceptibility artifacts. For liver measurements, ROIs were positioned in homogeneous regions of the hepatic parenchyma while excluding large blood vessels, bile ducts, and focal lesions. The average T2* values for both the heart and liver were recorded for subsequent analysis [19]. Lower T2* relaxation times were interpreted as indicating increased iron deposition within the respective organs.

#### 2.8.2. Serum Iron

In addition, SI, total iron-binding capacity (TIBC), and ferritin concentrations were measured using an automated clinical chemistry analyzer (Cobas 8000 modular series, Roche Diagnostics, Basel, Switzerland). SI and TIBC were determined using colorimetric assays, while ferritin levels were quantified using an electrochemiluminescence immunoassay method according to the manufacturer’s protocols [18]. For the analysis of SI, a Roche Iron Gen.2 reagent kit was used. TIBC was determined using the Roche UIBC reagent kit, which allowed for calculation of TIBC based on the measured UIBC values. Serum ferritin concentrations were measured using the Roche Elecsys Ferritin reagent kit. Calibration and internal quality control procedures were performed using Roche calibrators and control materials to ensure analytical accuracy and precision.

### 2.9. Oxidative Stress Assessment

#### 2.9.1. Antioxidant Capacity

Total antioxidant capacity of serum samples was determined using the ABTS radical cation (ABTS^•+^) decolorization assay, as previously described by Pellegrini et al. [20]. Briefly, the ABTS radical cation was generated by oxidizing 7 mM ABTS with 2.45 mM potassium persulfate. The resulting blue-green ABTS^•+^ stock solution was diluted with PBS, pH of 7.4, until an absorbance value of 0.7 at 734 nm was obtained. For the assay, 20 µL of the serum sample or Trolox standard solution (0.05–0.8 mg/mL), a water-soluble vitamin E analog, was mixed with 1.0 mL of the ABTS^•+^ working solution. The mixture was gently vortexed and incubated at room temperature for 6 min. The optical density (OD) was then measured at 414 nm against a reagent blank using a UV/visible double-beam spectrophotometer (Shimadzu Corporation, Nakagyo-ku, Kyoto, Japan). Antioxidant capacity was calculated relative to the Trolox standard curve and expressed in mg Trolox equivalent/mL (TEAC).

#### 2.9.2. GSH Concentrations

GSH concentrations were determined with a classical spectrophotometric method for redox biochemistry using Ellman’s reagent following the method established by Moron et al. [21]. The DTNB–GSH reaction generates the yellow product 5-mercapto-2-nitrobenzoic acid (TNB), allowing for the sensitive measurement of GSH. In the assay, serum was deproteinized with 25% trichloroacetic acid and centrifuged at 12,000 rpm (6900× *g*) at 4 °C for 10 min. Afterward, the supernatant was collected and incubated with Ellman’s reagent containing 0.2 M PB, pH 8.0, and 0.06 mM DTNB for 10 min at room temperature. Finally, the OD value of the colored product was measured at 412 nm against a reagent blank using a UV/visible double-beam spectrophotometer.

#### 2.9.3. TBARS Concentrations

Serum (80 µL) was mixed with 0.2% BHT (10 µL), 0.44 M H_3_PO_4_ (240 µL), and 0.6% TBA (160 µL). Subsequently, the mixture was incubated at 90 °C for 30 min, cooled down on ice, and the OD value was measured at 540 nm against the reagent blank using spectrophotometry [22].

### 2.10. Flow Cytometric Analysis

#### 2.10.1. RAI

Intracellular RAI was measured using the fluorescent probe FO, which irreversibly reacts with redox-active Fe^2+^. The assay was performed according to the method of Mei et al. [23] with minor modifications. FO (1 mM) was prepared in 50 mM HEPES buffer (pH 7.4). Cells were incubated with 2 µL of the FO working solution at 37 °C for 30 min. Fluorescence intensity (FI) was measured using a BD FACSAria III cell sorter (BD Biosciences, Milpitas, CA, USA) equipped with 488 nm, 561 nm, and 638 nm lasers. Emission was detected at 580 nm following excitation at 543 nm. Data acquisition and analysis were performed using BD FACSDiva software (version 9.0) on a Windows 10 (64-bit) system. Samples were measured at a low flow rate (10–20 µL/min) to minimize coincident events. Photomultiplier tube voltages were optimized using unstained and single-stained controls and maintained constant throughout each experiment. The gating strategy included sequential selection of cell populations based on forward scatter (FSC) and side scatter (SSC) to exclude debris, followed by singlet discrimination (FSC-A vs FSC-H) and the selection of viable cells, after which, FI was quantified in the corresponding detection channel. Acquisition was stopped after 10,000 viable singlet events per sample (or 20,000 events when sample parity was not required). Flow cytometry data were exported from BD FACSDiva version 9.0 and analyzed using FlowJo version 10 (Tree Star Inc., Ashland, OR, USA) or equivalent analysis software.

#### 2.10.2. Cellular ROS

Intracellular ROS levels were determined using H_2_DCFDA fluorochrome. After cellular uptake, the probe was hydrolyzed by intracellular esterases to H_2_DCF, which was oxidized by ROS to produce the fluorescent compound dichlorofluorescein (DCF). The assay was performed following the method of Amer et al. [24] with minor modifications. Treated cells were incubated with 9 µM H_2_DCFDA at 37 °C for 30 min in the dark. The FI of oxidized DCF was measured using the BD FACSAria III flow cytometer with excitation at 485 nm and emission at 525 nm. Data acquisition and analysis were performed using BD FACSDiva version 9.0 under the same acquisition settings described above.

#### 2.10.3. Membrane LPOs

Membrane leucocyte LPOs were assessed using LF, a fluorescent probe selective for lipid peroxides. The assay was conducted according to the method of Zheng et al. [25]. Briefly, 0.1 mL of leucocyte suspension was stained with 5 µL of 20 µM LF (prepared in 1% DMSO) and incubated at 37 °C for 15 min. Subsequently, 100 µM cumene hydroperoxide was added to induce lipid peroxidation, and cells were incubated at 37 °C for two hours. Cells were then washed twice with PBS. The FI was measured using the BD FACSAria III flow cytometer with excitation at 488 nm and emission at 535 nm. Data were acquired and analyzed using BD FACSDiva version 9.0.

### 2.11. Statistical Analysis

Statistical analysis was conducted using the IBM SPSS Statistics software, version 21.0 (IBM Corp., New York, NY, USA). Demographic characteristics were analyzed using descriptive statistical methods. Data are presented as mean ± standard deviation (SD) values for normally distributed variables or median values with interquartile ranges (IQRs) for non-normally distributed data. The normality of data distribution was assessed using the Shapiro–Wilk test. For hematological parameters, differences among the six study groups (healthy controls, IDA, obesity, TT, BTE, and BTM) were analyzed using one-way analysis of variance (ANOVA) followed by Tukey’s multiple comparison test when appropriate. For serum biochemical parameters, comparisons between each clinical group and the healthy controls were performed using an independent two-sample *t*-test. For iron metabolism parameters, statistical comparisons of groups were performed using one-way ANOVA or the Kruskal–Wallis test depending upon data distribution, followed by the appropriate post hoc multiple comparison analysis. For flow cytometric oxidative and functional markers, statistical differences between groups were evaluated using the Kruskal–Wallis test, followed by Dunn’s post hoc test with Holm adjustment for multiple comparisons. For oxidative stress biomarkers, group comparisons were performed using the Kruskal–Wallis test, followed by pairwise Mann–Whitney U tests with Bonferroni corrections. Statistical significance is indicated as * *p* < 0.05, ** *p* < 0.01, and *** *p* < 0.001. The complete anonymized dataset generated and analyzed during this study is provided as a supplementary spreadsheet (Supplementary Materials) to allow for independent verification and secondary analyses.

## 3. Results

### 3.1. Demographic and Clinical Characteristics of Study Participants

Demographic and clinical characteristics, and treatments of non-thalassemic and thalassemia participants are shown in Table 1 and Table S1. The 18 non-thalassemic subjects included healthy controls, as well as individuals with IDA and obesity. Among the healthy control participants (n = 14), the mean age was 35.6 ± 8.0 years, with a predominance of females (12 females and 2 males). None of the control subjects had a history of blood transfusions (BTX) or a splenectomy (SPX), and none had received iron chelation therapy. Two participants were diagnosed with iron IDA (mean age of 35.0 ± 5.7 years), both female. Similar to the healthy controls, these individuals had no history of BTX or SPX and were not receiving any iron chelators such as desferrioxamine (DFO), deferiprone (DFP) or deferasirox (DFX). Additionally, two subjects with obesity were included; the mean age was 43.5 ± 20.5 years. These participants also showed no history of having recently undergone a transfusion, splenectomy, or iron chelation therapy. Overall, the non-thalassemic group consisted mainly of healthy individuals without clinical evidence of iron overload or treatment interventions related to iron metabolism.

Thalassemia patients included those diagnosed with TT, BTE, and BTM. Two participants with TT were included (mean age of 49.5 ± 7.8 years), both female. Neither participant had a history of undergoing a blood transfusion or a splenectomy, and no iron chelation therapy was reported. The largest patient subgroup consisted of BTE patients (n = 28; mean age of 38.9 ± 13.6 years), which included 13 males and 15 females. All individuals in this group had a history of blood transfusions, while splenectomies were reported in 12 patients. Various iron chelation therapies were used, including DFP, DFX, DFO, and combination therapy, reflecting ongoing clinical management of transfusion-related iron overload. Six BTM patients were included (mean age of 31.8 ± 6.8 years): 2 males and 4 females. All BTM patients had received blood transfusions, and five out of six patients had undergone a splenectomy. Most patients in this group received iron chelation therapy, predominantly combination therapy, or DFO-based treatment. Collectively, these data demonstrate clear clinical differences between control participants and thalassemia patients. While non-thalassemic individuals showed no evidence of transfusion exposure or iron-related treatment, thalassemia patients, particularly BTE and BTM patients, commonly required blood transfusions and/or iron chelation therapy.

### 3.2. Hematological Characteristics of Study Participants

**Figure 1:**
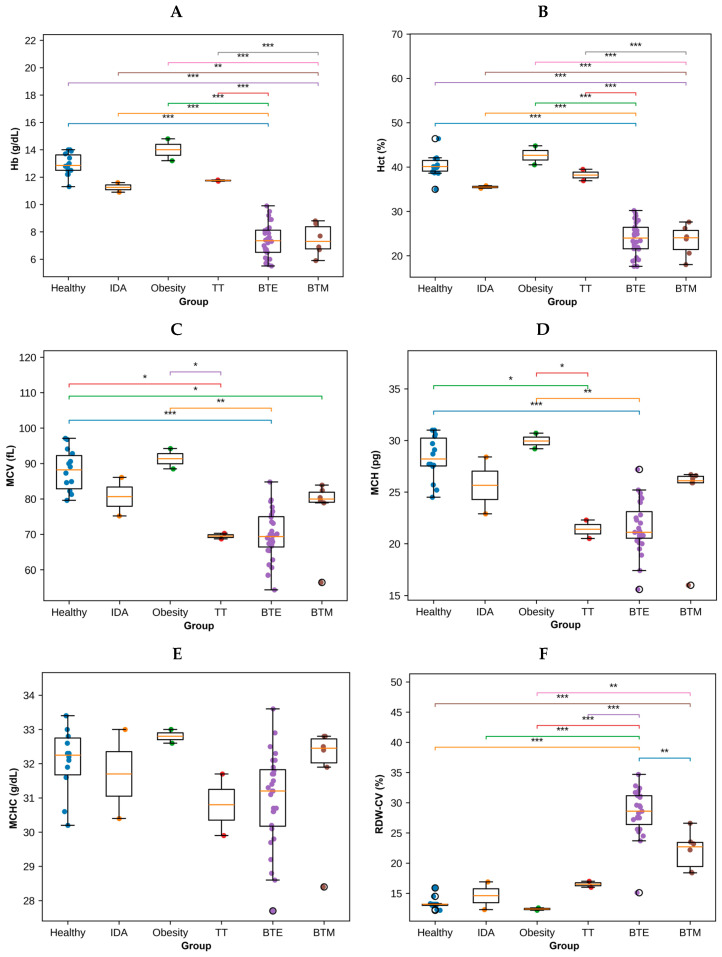
RBC parameters in healthy individuals (n = 14) and patients with IDA (n = 2), obesity (n = 2), TT (n = 2), BTE (n = 28) and BTM (n = 6). Boxplots showing hematological RBC indices including (**A**) Hb, (**B**) Hct, (**C**) MCV, (**D**) MCH, (**E**) MCHC, and (**F**) RDW-CV in study groups. Individual dots represent measurements from each participant. Boxes indicate the IQRs, horizontal lines represent the medians, and whiskers indicate the minimum and maximum values. Statistical differences among groups were assessed using one-way ANOVA followed by Tukey’s multiple comparison test. * *p* < 0.05, ** *p* < 0.01, *** *p* < 0.001 indicate statistically significant differences.

Significant differences in RBC indices were observed among the six study groups: healthy controls, IDA, obesity, TT, BTE, and BTM (Figure 1 and Table S2). Hb levels varied significantly across all groups (*p* < 0.001). The highest Hb concentrations were observed in the obesity group, followed by the healthy controls. In contrast, markedly reduced Hb levels were detected in the BTE and BTM groups, indicating severe anemia. The individuals with IDA and TT showed intermediate Hb values but they remained significantly lower than those of the healthy subjects. A similar pattern was observed for Hct, which was significantly decreased in the BTE and BTM groups compared with all the other groups (*p* < 0.001). The obesity group demonstrated the highest median Hct values, while the IDA and TT groups exhibited moderate reductions relative to healthy controls. Mean corpuscular volume (MCV) differed significantly among groups (*p* < 0.001). The lowest MCV values were observed in TT and BTE patients, indicating pronounced microcytosis. The IDA group also demonstrated reduced MCVs when compared with healthy individuals, while the obesity group maintained values within the normal range. Mean corpuscular hemoglobin (MCH) followed a similar trend, with significantly lower levels in the TT and BTE groups when compared with the healthy controls (*p* < 0.001). The IDA group showed moderately reduced MCH values, whereas the healthy and obesity groups exhibited the highest values. In contrast, mean corpuscular hemoglobin (MCHC) displayed only minor variations between groups and did not show substantial group separation. Red blood cell distribution width coefficient of variation (RDW-CV) differed significantly between groups (*p* < 0.001). The highest RDW values were observed in BTE and BTM subjects, reflecting increased variability in erythrocyte size. The IDA group also demonstrated elevated RDW values relative to the healthy controls, while the obesity group showed the lowest RDW values.

**Figure 2:**
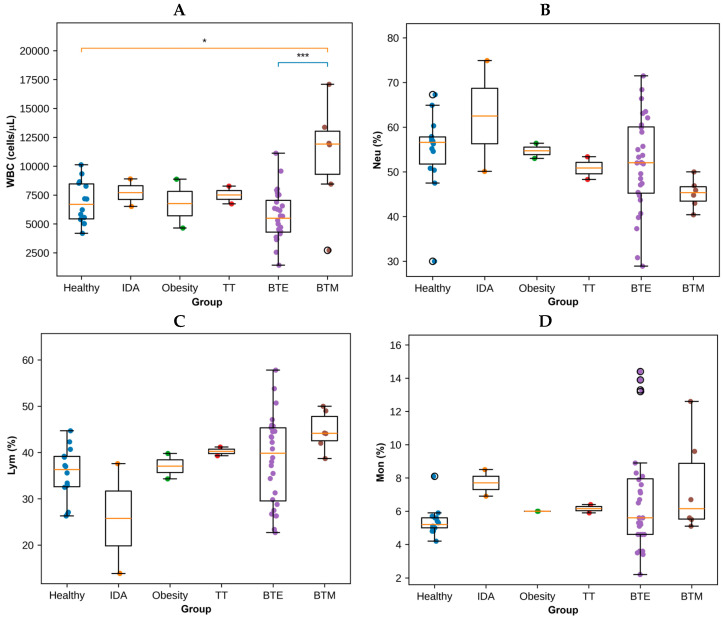
WBC number and differential parameters in healthy individuals and patient groups. Boxplots illustrating (**A**) total WBC, (**B**) Neu percentage, (**C**) Lym percentage, and (**D**) Mon percentage in healthy controls and patient groups. * *p* < 0.05, *** *p* < 0.001 indicate significant differences between groups.

WBC counts also differed among study groups (Figure 2 and Table S2). The BTM group exhibited the highest WBC counts, while the BTE group showed a wide distribution with considerable variability. The healthy, IDA, obesity, and TT groups displayed relatively comparable WBC ranges. An analysis of leukocyte differentials revealed variations in neutrophil (Neu), lymphocyte (Lym), and monocyte (Mon) percentages across groups. Neutrophil percentages were generally higher in the IDA group compared with the other groups, whereas the BTE group exhibited substantial variability. Lymphocyte percentages were highest in the BTM group and lowest in the IDA group. Monocyte levels showed modest differences across groups but displayed greater variability within the BTE group.

**Figure 3:**
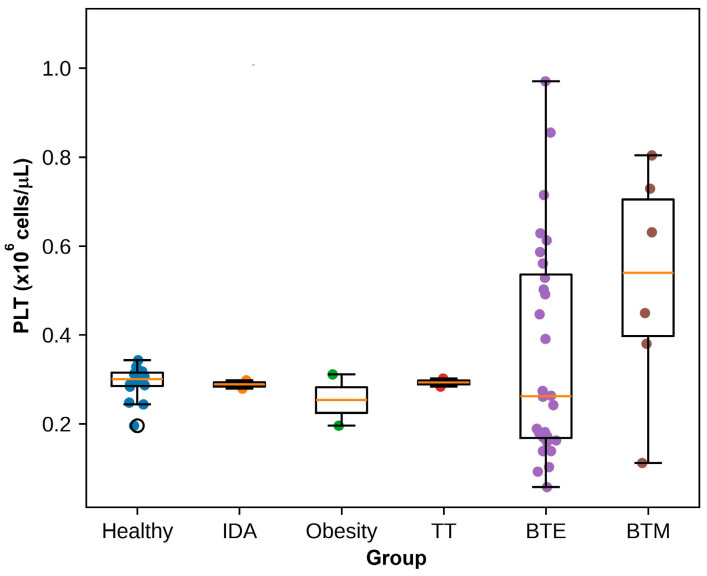
PLT counts in healthy individuals and patient groups. Boxplot showing PLT in healthy controls and patients with IDA, obesity, TT, BTE, and BTM.

PLT counts varied considerably among the groups (Figure 3 and Table S2). The healthy controls, IDA, obesity, and TT groups showed relatively narrow distributions with similar median values. In contrast, the BTE and BTM groups demonstrated markedly wider ranges of platelet counts, indicating substantial inter-individual variability within these conditions. The BTM group exhibited the highest upper range of platelet counts among all groups.

Overall, multiple hematological parameters, particularly Hb, Hct, MCV, MCH, and RDW, showed statistically significant differences between the studied groups, highlighting the distinct hematological profiles associated with iron deficiency anemia, obesity, and thalassemia conditions.

### 3.3. Serum Biochemical Parameters

**Figure 4:**
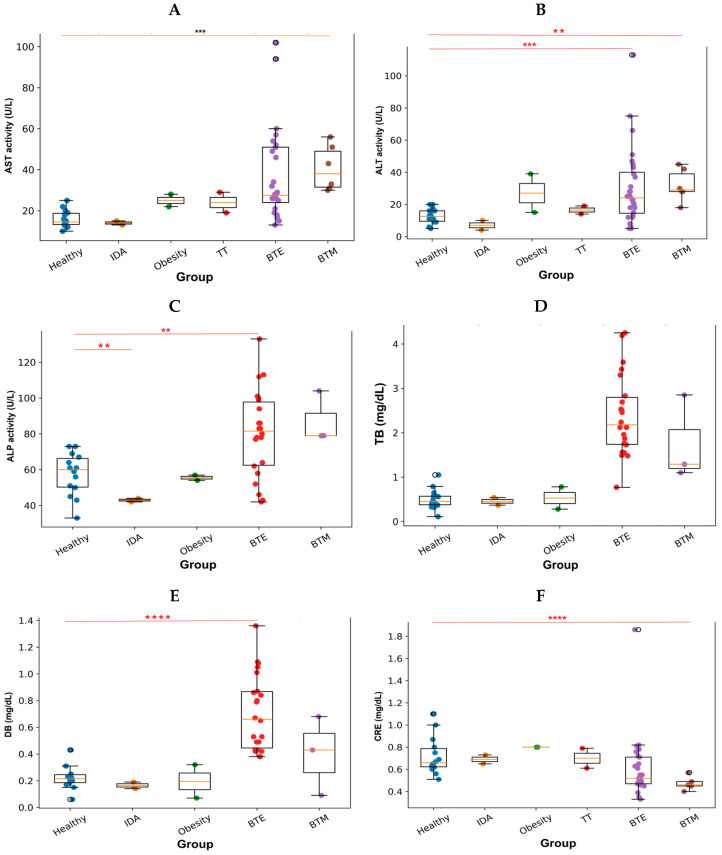
Serum biochemical parameters of study groups. Boxplots represent the (**A**) AST, (**B**) ALT, (**C**) ALP, (**D**) TB, (**E**) DB, and (**F**) CRE levels in the healthy controls, IDA, obesity, TT, BTE, and BTM groups. Boxes indicate the IQR with the median shown as a horizontal line. Whiskers represent the range of the data excluding outliers. Individual data points are shown as dots. Statistical comparisons between each group and healthy controls were performed using an independent two-sample *t*-test, and the corresponding *p*-values are indicated as follows: ** *p* < 0.01; *** *p* < 0.005, **** *p* <0.001.

Serum biochemical parameters were analyzed in the study participants. These included liver enzymes, such as AST, ALT and ALP, total bilirubin (TB), direct bilirubin (DB), and creatinine (CRE) levels (Figure 4 and Table S3). Healthy individuals showed normal ranges of biochemical parameters, with mean AST, ALT, and ALP activities of 16.0 ± 4.3 U/L, 12.9 ± 4.8 U/L, and 57.5 ± 11.9 U/L, respectively. The mean TB and DB levels were 0.50 ± 0.23 and 0.23 ± 0.09 mg/dL, respectively, while serum CRE was 0.72 ± 0.17 mg/dL. The individuals with IDA demonstrated biochemical values comparable to the healthy controls, with mean AST and ALT levels of 14.0 ± 1.4 U/L and 7.0 ± 4.2 U/L, respectively, and mean CRE levels of 0.69 ± 0.06 mg/dL, suggesting preserved liver and renal function in this group.

In contrast, the patients with β-thalassemia showed notable elevations in several biochemical markers. The BTE group exhibited increased liver enzyme activities with mean AST and ALT levels of 37.2 ± 22.3 U/L and 30.4 ± 24.0 U/L, respectively, which were substantially higher than those observed in the healthy controls. Additionally, ALP activity was elevated in BTE patients (80.4 ± 24.1 U/L). Total bilirubin levels were also markedly increased in this group (2.38 ± 0.91 mg/dL), indicating enhanced hemolysis associated with thalassemia pathology. Similarly, the BTM group showed elevated liver enzyme activities, with mean AST and ALT levels of 40.7 ± 11.1 U/L and 31.8 ± 10.0 U/L, respectively. TB levels were also increased (1.75 ± 0.96 mg/dL), reflecting increased erythrocyte destruction and hemolytic activity in these patients. ALP levels were slightly higher when compared with the healthy controls (87.3 ± 14.4 U/L). Serum CRE levels remained relatively stable across most groups, with mean values of 0.61 ± 0.29 mg/dL in BTE patients and 0.47 ± 0.06 mg/dL in BTM patients, suggesting that renal function was largely preserved in the studied cohort. Thus, the biochemical findings indicate that thalassemia patients, particularly those with β-thalassemia HbE and β-thalassemia major, exhibit elevated liver enzyme activities and bilirubin levels compared with healthy individuals, reflecting increased hemolysis and possible hepatic involvement associated with chronic transfusion and iron overload.

### 3.4. Iron Status Parameters

#### 3.4.1. Heart and Liver Iron Accumulation

**Figure 5:**
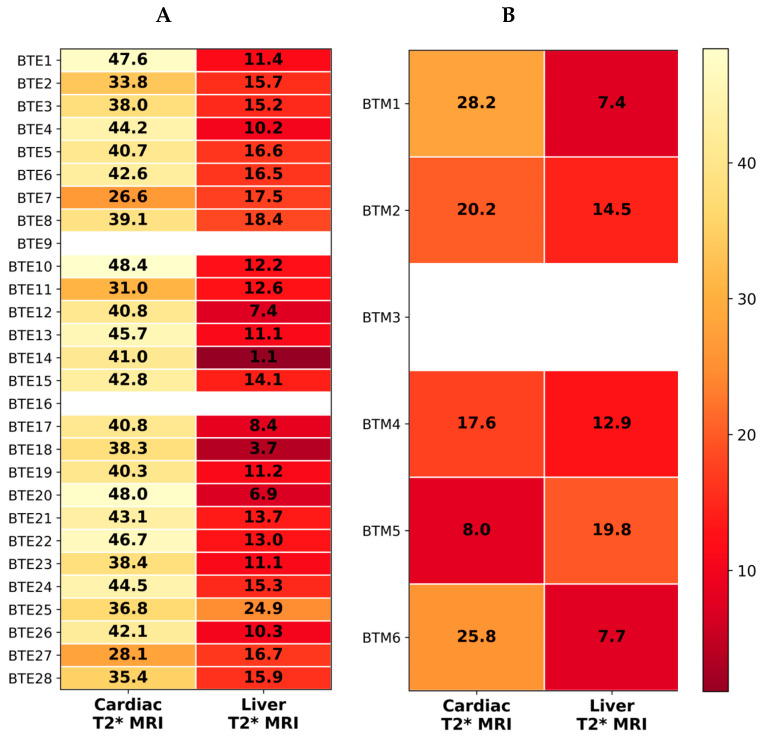
Heatmap visualization of cardiac and liver iron burden measured by T2* MRI technique in (**A**) BTE patients (n = 26) and (**B**) BTM patients (n = 5). Each row represents a patient, and each column represents the T2* MRI measurement for the heart or liver. Values within cells indicate the measured T2* MRI relaxation time (ms). Color intensity corresponds to T2* values, where lower T2* values indicate greater iron accumulation.

The clinical interpretation of the T2* values follow established thresholds used in non-invasive studies of iron overload in thalassemia patients: cardiac T2* >20 ms is normal, 15–20 ms is mild, 10–14.9 ms is moderate, and <10 ms is severe, whereas liver T2* >15.4 ms is normal, 4.5–15.4 ms is mild, 2.1–4.4 ms is moderate, and <2.1 ms is severe. β-Thalassemia patients, but not healthy controls, were requested to receive T2* MRI treatment. The heatmap analysis revealed distinct patterns of cardiac and liver iron accumulation between BTE and BTM patients. In the BTE group, cardiac T2* values were generally within the normal range (>20 ms), indicating minimal myocardial iron deposition in most individuals with a mean ± SD value of 40.18 ± 5.73 ms, whereas hepatic T2* values were frequently reduced to that of 12.73 ± 4.91 ms (Figure 5A). This would suggest mild to moderate hepatic iron overload, which is consistent with preferential iron accumulation in the liver during early or less severe transfusion exposure. In contrast, BTM patients demonstrated greater heterogeneity in cardiac iron burden (19.96 ± 7.92 ms) and hepatic iron burden (19.96 ± 5.16 ms) (Figure 5B). While several patients maintained normal cardiac T2* values, one patient exhibited markedly reduced cardiac T2* (<10 ms), indicating severe myocardial iron deposition, a known complication of transfusion-dependent thalassemia. Hepatic iron loading was also evident in several BTM patients, with T2* values consistent with mild to moderate liver iron overload. Overall, these findings highlight that hepatic iron accumulation occurs more frequently and earlier than cardiac iron deposition, whereas severe myocardial iron overload appears predominantly in patients with β-thalassemia major, reflecting the cumulative effects of chronic transfusion therapy and systemic iron loading.

#### 3.4.2. Plasma Iron Levels

**Figure 6:**
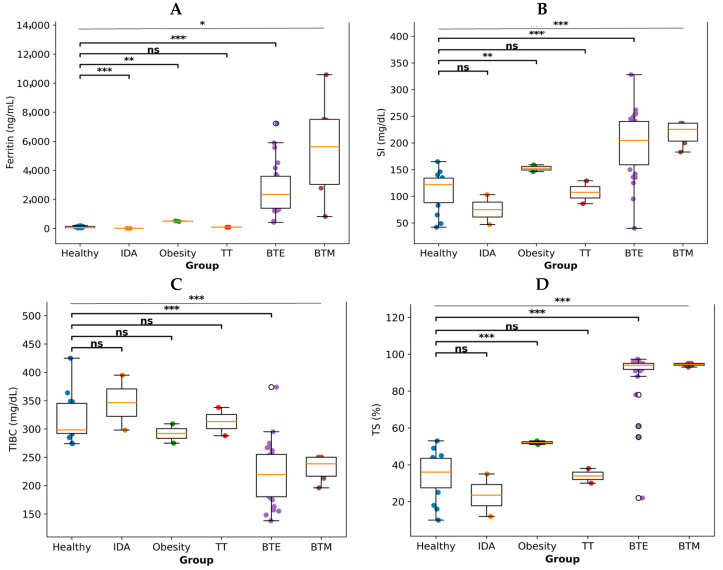
Iron metabolism parameters in healthy controls and thalassemia groups. Boxplots show the levels of (**A**) ferritin, (**B**) SI, (**C**) TIBC, and (**D**) TS in healthy control, IDA, obesity, TT, BTE and BTM groups. Boxes indicate the IQR, the horizontal line within each box indicates the median, and whiskers represent the data range excluding outliers. Individual values are shown as colored dots. Statistical significance versus healthy controls is indicated as not significant (ns); * *p* < 0.05; ** *p* < 0.01; *** *p* < 0.001.

Plasma iron parameters also differed among the clinical groups (Figure 6). Ferritin, SI, TIBC, and TS revealed distinct distributions across the healthy control, IDA, obesity, TT, BTE, and BTM groups. Thalassemia patients generally showed elevated ferritin and transferrin saturation levels compared with healthy controls, indicating systemic iron overload, whereas IDA subjects displayed patterns consistent with reduced iron availability. Together, these findings demonstrate preferential hepatic iron accumulation in BTE and greater variability in cardiac and hepatic iron loading in BTM, accompanied by significant alterations in circulating iron metabolism markers.

### 3.5. Comparison of Oxidative and Functional Markers in Granulocytes and Lymphocytes in Clinical Groups

**Figure 7:**
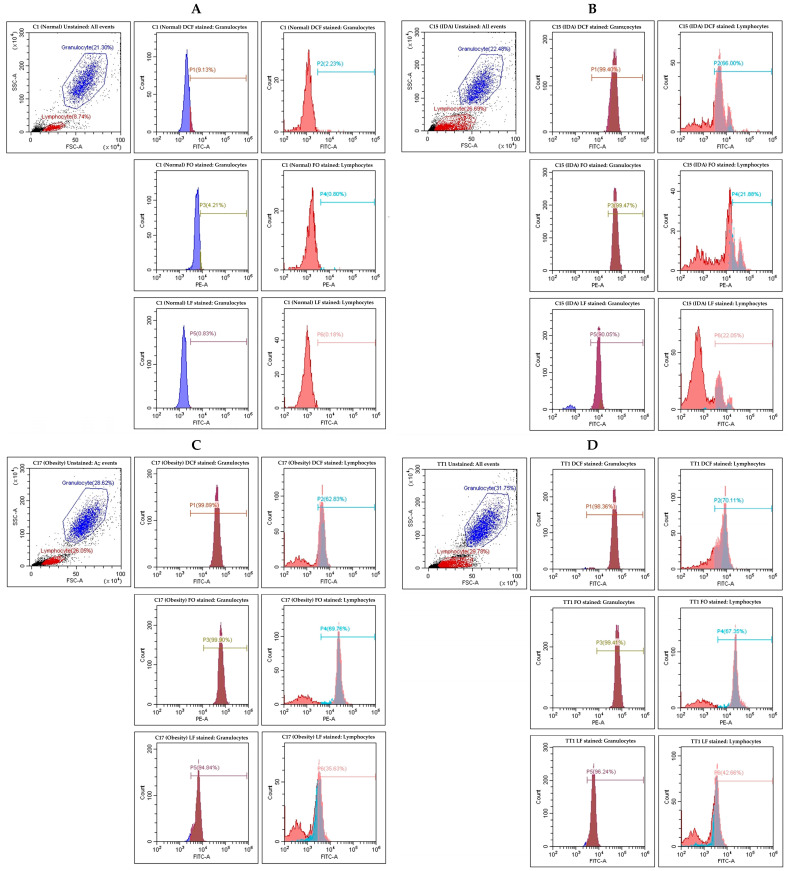

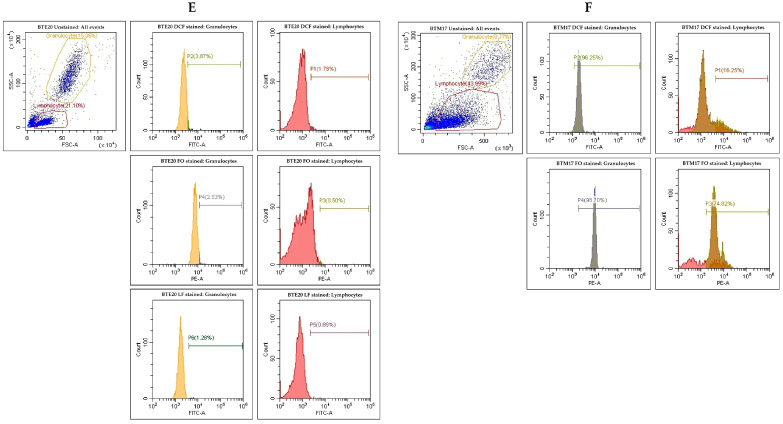
Representative flow cytometric histograms of granulocytes and lymphocytes stained with oxidative stress markers in different clinical groups: (**A**) Normal group, (**B**) IDA group, (**C**) Obesity group, (**D**) TT group, (**E**) BTE group, and (**F**) BTM group.

Representative flow cytometric histograms illustrating the distribution of FI in granulocytes and lymphocytes from individuals belonging to different clinical groups are shown in Figure 7. The healthy controls exhibited strong fluorescence signals (Figure 7A), indicating normal intracellular oxidative activity and intact immune cell function. The samples from individuals with IDA (Figure 7B) and obesity (Figure 7C) showed modest variations in FI, suggesting mild alterations in oxidative metabolism associated with these metabolic conditions. Individuals with TT displayed fluorescence patterns (Figure 7D) comparable to the healthy controls, suggesting that mild genetic alterations in Hb synthesis do not substantially affect immune cell oxidative responses. In contrast, the samples from patients with BTE (Figure 7E) and BTM (Figure 7F) demonstrated altered fluorescence distributions, particularly in granulocyte populations, indicating impaired oxidative burst activity. These representative histograms support the quantitative findings that oxidative and functional alterations were most pronounced in granulocytes obtained from β-thalassemia patients, whereas lymphocyte responses remained relatively preserved.

**Figure 8:**
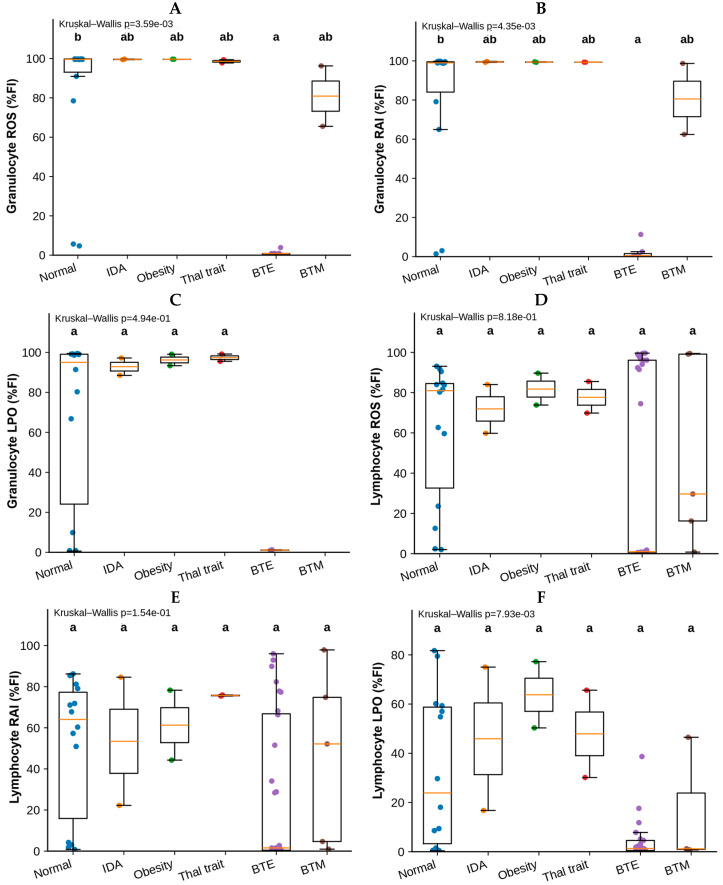
Comparison of granulocyte and lymphocyte functional markers in clinical groups. Boxplots show the FI for ROS, RAI, and LPOs in granulocytes (**A**–**C**) and lymphocytes (**D**–**F**) in the six study groups: healthy control, IDA, obesity, TT, BTE and BTM. Individual data points represent measurements from each participant. The central line within each box represents the median, the box indicates the IQR, and whiskers represent the distribution of the data excluding outliers. Statistical differences among groups were evaluated using the Kruskal–Wallis test, followed by Dunn’s post hoc multiple comparison test with Holm adjustments. Groups sharing at least one identical letter above the boxplots are not significantly different, whereas groups with different letters differ significantly (adjusted *p* < 0.05).

The distribution of ROS, RAI, and LPO fluorescence intensity (%FI) in granulocytes and lymphocytes in the study groups (healthy control, IDA, obesity, thalassemia trait, BTE, and BTM) is shown in Figure 8A–F. Statistical comparisons were performed using the Kruskal–Wallis test, followed by Dunn’s post hoc test with Holm adjustments for multiple comparisons. Groups sharing identical letters in the figure are not significantly different.

Granulocyte ROS levels differed significantly among the study groups (Kruskal–Wallis *p* = 3.59 × 10^−3^) (Figure 8A). The healthy controls demonstrated high ROS levels, reflecting robust oxidative burst activity. In contrast, the BTE samples showed markedly reduced ROS levels, indicating impaired granulocyte oxidative activity. The BTM samples displayed intermediate values, while the IDA, obesity, and TT groups exhibited values comparable to the healthy controls. Post hoc analysis indicated that the BTE group differed significantly from the healthy group, whereas the other groups were not significantly different. A similar pattern was observed for granulocyte RAI (Kruskal–Wallis *p* = 4.35 × 10^−3^) (Figure 8B). The healthy, IDA, obesity, and TT groups demonstrated consistently high RAI values. In contrast, the BTE samples exhibited markedly lower RAI levels, suggesting substantial functional impairment of granulocytes. The BTM samples showed moderate reductions but remained closer to healthy levels than the BTE samples. Dunn’s post hoc analysis confirmed that the BTE group differed significantly from healthy controls, whereas the other groups did not show statistically significant differences. No statistically significant differences were observed for granulocyte LPOs among the groups (Kruskal–Wallis *p* = 0.49) (Figure 8C). The FI of LPOs remained relatively consistent across all clinical conditions, suggesting that this marker may be less sensitive to disease-related alterations in granulocyte function.

In comparison, lymphocyte ROS levels did not differ significantly among the study groups (Kruskal–Wallis *p* = 0.818) (Figure 8D). Although individual variability was observed, the median FI remained comparable across all groups, indicating that lymphocyte oxidative activity was largely preserved across these clinical conditions. Similarly, lymphocyte RAI showed no statistically significant differences between groups (Kruskal–Wallis *p* = 0.154) (Figure 8E). While variability in FI was observed, no consistent disease-associated pattern emerged. In contrast, lymphocyte LPO levels differed significantly among the groups (Kruskal–Wallis *p* = 7.93 × 10^−3^) (Figure 8F). The healthy control, IDA, obesity, and TT groups demonstrated relatively higher LPO levels. However, the BTE and BTM groups showed lower LPO levels, suggesting altered lymphocyte functional responses in individuals with more severe forms of β-thalassemia. Taken together, these results indicate that granulocyte oxidative burst activity (ROS and RAI) was significantly altered in the BTE group, whereas granulocyte LPO activity remained relatively stable across groups. In contrast, lymphocyte markers showed limited group-dependent differences, with the exception of LPOs, which appeared to be reduced in severe β-thalassemia conditions. These findings suggest that granulocyte functional impairment may be a more prominent feature of disease-associated immune dysregulation than lymphocyte dysfunction in these patient populations. Significant differences were observed in granulocyte ROS and RAI, indicating altered granulocyte oxidative activity among clinical groups, particularly in β-thalassemia conditions. Lymphocyte LPO levels also showed significant group differences, whereas granulocyte LPOs, lymphocyte ROS, and lymphocyte RAI did not show statistically significant variations across groups.

### 3.6. Oxidative Stress Biomarkers Across Clinical Groups

**Figure 9:**
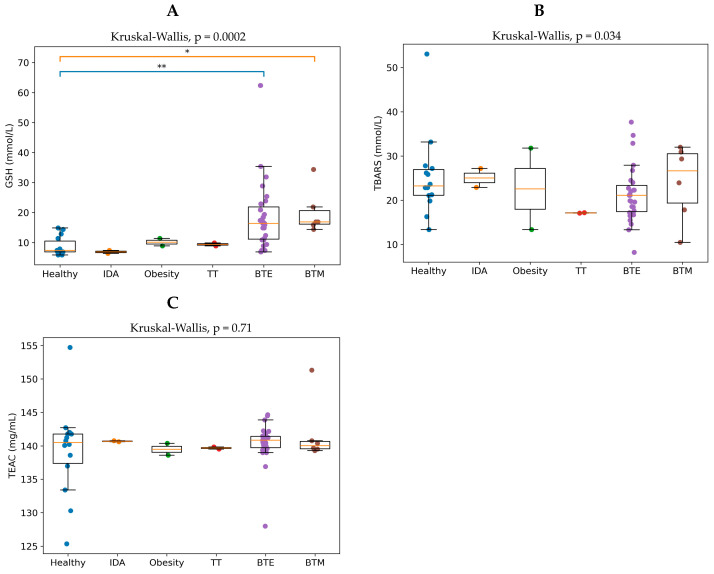
GSH (**A**), TBARS (**B**), and TEAC (**C**) levels in different clinical groups. Boxplots illustrate the GSH, TBARS, and AC levels measured in peripheral blood samples obtained from individuals belonging to the healthy control (n = 14), IDA (n = 2), obesity (n = 2), TT (n = 2), BTE (n = 28), and BTM (n = 6) groups. Each dot represents an individual subject, while the box represents the IQR and the central line indicates the median value. Whiskers represent the spread of the data excluding outliers. Statistical comparisons of groups were performed using the Kruskal–Wallis test, followed by pairwise Mann–Whitney tests with Bonferroni correction. Overall group differences were evaluated using the Kruskal–Wallis test, which showed a significant difference among groups (*p* = 2.03 × 10^−4^). * *p* < 0.05 and ** *p* < 0.01 indicate significant differences between groups.

The analysis showed significant differences in GSH levels among the clinical groups (Kruskal–Wallis *p* ≈ 2.03 × 10^−4^) (Figure 9A). The healthy controls displayed moderate GSH levels with relatively low variability, indicating normal antioxidant status. The subjects with IDA showed slightly lower GSH levels, which may reflect the reduced antioxidant capacity that is associated with impaired iron metabolism. The individuals with obesity and TT exhibited GSH levels similar to those of the healthy controls, suggesting relatively preserved antioxidant defense mechanisms in these groups. In contrast, the BTE group demonstrated a wide distribution and higher variability of GSH values, with some individuals showing markedly elevated levels. This pattern likely reflects compensatory upregulation of glutathione production in response to the chronic oxidative stress caused by ineffective erythropoiesis and iron overload. The patients with BTM also showed elevated GSH levels when compared with the healthy individuals, although the variability was lower than that which was observed in the BTE group.

However, the statistical analysis revealed no significant differences in serum TBARS levels among the clinical groups, indicating that the degree of lipid peroxidation was relatively similar across the study population (Figure 9B). Although thalassemia is known to be associated with oxidative stress due to iron overload and chronic hemolysis, the absence of significant differences in TBARS levels may suggest that lipid peroxidation was either well controlled by antioxidant defenses or not substantially elevated in this cohort. Likewise, the results showed no significant differences in TEAC values between the clinical groups (Figure 9C), indicating that total antioxidant capacity remained relatively stable across the study population. This finding suggests that although individual antioxidant components, such as GSH, may vary, the overall antioxidant defense system may remain balanced through compensatory mechanisms. Taken together, the results indicate that changes in oxidative balance among the clinical groups are primarily reflected in intracellular antioxidant mechanisms rather than in global oxidative damage markers. Significant variations in GSH levels suggest that glutathione plays a key role in the antioxidant response to oxidative stress, particularly in β-thalassemia conditions. In contrast, the lack of significant differences in TBARSs and TEAC would indicate that overall lipid peroxidation and total antioxidant capacity remain relatively stable among the groups. These findings highlight the importance of glutathione-mediated antioxidant defense mechanisms in maintaining redox homeostasis in individuals with thalassemia and related conditions.

## 4. Discussion

Thalassemia is characterized by significant alterations in hematological parameters, iron metabolism, oxidative stress, and immune function. In the present study, we investigated these parameters across several clinical groups, including healthy individuals and patients with IDA, obesity, TT, BTE, and BTM. Our findings demonstrate substantial differences in hematological profiles, biochemical parameters, iron metabolism indicators, oxidative stress biomarkers, and immune cell functional responses among these groups, with the most pronounced alterations observed in patients with β-thalassemia. As expected, significant abnormalities in RBC indices were observed in thalassemia patients. Reduced Hb, Hct, MCV, and MCH values in the BTE and BTM groups reflect the microcytic hypochromic anemia characteristic of thalassemia, which can result from globin chain imbalance and ineffective erythropoiesis [3,4]. Elevated RDW values further indicate increased variability in erythrocyte size caused by abnormal erythrocyte production and destruction. Biochemical analysis also revealed elevated liver enzyme activities and bilirubin levels in thalassemia patients, particularly in the BTE and BTM groups. These findings suggest the hepatic involvement associated with chronic hemolysis and transfusion-related iron overload. Previous studies have shown that repeated transfusions and iron deposition in hepatic tissue contribute to hepatocellular injury and metabolic disturbances in thalassemia patients [3,5].

Alterations in plasma liver enzymes, such as AST, ALT, and ALP, are commonly associated with oxidative stress and metabolic dysfunction, particularly in conditions characterized by iron imbalance or metabolic disorders [8,26,27]. Thalassemia patients frequently exhibit elevated liver enzyme activities and bilirubin levels, reflecting the hepatic stress associated with chronic hemolysis, transfusion-related iron overload, and oxidative injury [1,2,28]. This study revealed elevated liver enzyme activities and bilirubin levels in thalassemia patients, particularly in the BTE and BTM groups. The increased AST, ALT, and ALP levels suggest hepatic stress or damage, which may have resulted from chronic hemolysis, repeated BTX, and iron accumulation in the liver. Elevated bilirubin levels further support increased erythrocyte destruction, a hallmark of thalassemia pathophysiology [1].

The assessment of tissue iron accumulation using T2* MRI demonstrated distinct patterns of iron distribution between BTE and BTM patients. In the BTE patients, cardiac T2* values were largely within the normal range, whereas hepatic T2* values frequently indicated mild to moderate iron overload. In contrast, the BTM patients showed greater variabilities in cardiac iron burden, including cases of severe myocardial iron deposition. Distinct iron accumulation patterns and oxidative stress findings were observed in the BTE group while remaining comparable with the BTM group, reflecting the integrative design of the study. These findings are consistent with those of previous studies demonstrating that iron accumulation occurs earlier and more extensively in the liver, while cardiac iron overload tends to develop later in transfusion-dependent thalassemia patients [10,11]. Disturbances in plasma iron parameters were also evident in the present study. The elevated ferritin levels and increased transferrin saturation observed in the thalassemia patients indicate systemic iron overload resulting from both transfusion therapy and increased intestinal iron absorption associated with ineffective erythropoiesis [4,29]. Excess iron promotes the formation of reactive oxygen species through redox reactions, leading to oxidative stress and tissue damage. Oxidative stress plays a critical role in the pathophysiology of thalassemia. Increased oxidative stress in thalassemia has been attributed to several mechanisms, including iron-mediated generation of reactive oxygen species, chronic hemolysis, and ineffective erythropoiesis [9]. These processes contribute to oxidative damage in erythrocytes and other tissues. In the present study, significant variations in glutathione levels were observed across clinical groups, suggesting activation of antioxidant defense mechanisms in response to chronic oxidative stress. Glutathione is an essential intracellular antioxidant that protects cells from oxidative damage, and increased levels may represent a compensatory response aimed at maintaining redox balance [12,13].

In addition to systemic oxidative stress, our results revealed alterations in immune cell function. Flow cytometric analysis demonstrated significantly reduced granulocyte oxidative burst activity in the BTE patients, whereas lymphocyte oxidative responses remained largely unchanged. Neutrophils rely on the oxidative burst mechanism to generate the reactive oxygen species necessary for pathogen elimination; therefore, impaired oxidative burst activity may compromise innate immune responses. Previous studies have also reported abnormalities in neutrophil function and immune dysregulation in thalassemia patients, potentially contributing to increased susceptibility to infections [10,16]. Recently, Asadov and Aliyeva have reported the interplay between iron overload, oxidative stress, and immune cell dysfunction in β-thalassemia patients, providing insights that may support improved monitoring and therapeutic strategies for managing disease-associated oxidative damage and immune alterations [17]. Interestingly, lymphocyte oxidative markers showed fewer group-dependent differences. Lymphocyte ROS and RAI levels were largely comparable across the clinical groups, suggesting that lymphocyte oxidative responses remain relatively preserved. However, lymphocyte LPO levels were significantly reduced in the BTE and BTM groups, which may reflect altered cellular metabolism or oxidative signaling pathways in severe thalassemia conditions. IDA has also been associated with increased oxidative stress and altered antioxidant defense mechanisms. Studies have reported changes in lipid peroxidation markers, including malondialdehyde and TBARSs, as well as alterations in antioxidant enzymes and glutathione levels, indicating disruption of redox homeostasis in iron-deficient individuals [30,31,32]. Moreover, obesity is characterized by chronic oxidative stress resulting from excessive lipid accumulation, mitochondrial dysfunction, and low-grade inflammation. These processes lead to increased production of ROS and alterations in antioxidant capacity, including changes in GSH levels and total antioxidant capacity [33,34].

The analysis of systemic oxidative stress biomarkers further revealed significant differences in GSH levels across groups. Elevated and highly variable GSH levels in the BTE group likely reflect compensatory activation of antioxidant defense mechanisms in response to chronic oxidative stress. Increased oxidative stress in thalassemia has been attributed to several mechanisms, including iron-mediated generation of ROS, chronic hemolysis, and ineffective erythropoiesis, all of which contribute to cellular oxidative damage and disease progression [2,9,35]. Alterations in serum GSH and total antioxidant capacity may reflect adaptive responses aimed at counteracting oxidative damage. Such compensatory mechanisms have been observed in metabolic disorders and conditions involving iron imbalance, where antioxidant systems are activated to maintain cellular redox balance [14,15]. In contrast, TBARS and TEAC levels did not differ significantly among groups, suggesting that global oxidative damage and total antioxidant capacity may remain relatively stable, possibly due to adaptive antioxidant responses.

The heme oxygenase (HO) system likely plays a critical role in thalassemia, a condition characterized by chronic hemolysis and transfusion-related iron overload, both of which contribute to excess heme and iron accumulation [5,8]. Increased HO activity may represent an adaptive response to elevated intracellular heme levels [36,37]; however, excessive HO-mediated heme degradation can further increase the labile iron pool, thereby enhancing ROS generation through redox cycling reactions [9]. This mechanism is consistent with the elevated oxidative stress markers observed in our thalassemia cohorts. In particular, increased redox-active iron may promote lipid peroxidation and depletion of antioxidant defenses, as reflected by alterations in glutathione and related biomarkers [12,13]. Beyond its enzymatic products, the HO-mediated reduction of intracellular heme may also disrupt the heme-dependent signaling pathways [36] that regulate cellular homeostasis [14,15]. Such alterations may contribute to impaired immune cell function, particularly with regard to the reduced granulocyte oxidative burst activity observed in this study [16,17]. In contrast, these relatively preserved lymphocyte responses suggest the differential degrees of sensitivity of the immune cell subsets to chronic redox imbalance.

In parallel, the bone marrow microenvironment, a primary site of hematopoiesis and a dynamic cellular niche, may be profoundly affected by iron dysregulation. Specialized VCAM1^+^CD163^+^CCR3^+^ macrophages normally support erythropoiesis by supplying iron to erythroblasts; however, under iron-overloaded conditions, dysregulated macrophage iron handling may impair this process [38]. In thalassemia, excess labile iron and oxidative stress may disrupt macrophage-mediated iron trafficking, thereby limiting effective erythropoiesis and contributing to persistent anemia. Furthermore, altered macrophage function may promote a pro-oxidative microenvironment that exacerbates redox imbalance and immune dysfunction. Collectively, these findings suggest that disruption of the heme–HO axis, together with impaired macrophage-driven iron distribution in the bone marrow niche, represents a key mechanism linking iron overload, oxidative stress, ineffective erythropoiesis, and immune dysregulation in thalassemia.

Ferroptosis, an iron-dependent form of regulated cell death, is tightly regulated by intracellular iron availability, ROS generation, and antioxidant systems (e.g., GSH), all of which are highly relevant to the pathological features observed in this study. In cases of thalassemia, chronic iron overload and increased labile iron may promote lipid peroxidation and sensitize cells to ferroptotic damage, consistent with our findings of altered glutathione levels and increased oxidative stress markers. In addition, ferroptosis has been implicated in immune regulation and inflammatory responses, suggesting that redox imbalance may further contribute to impaired granulocyte function. Emerging evidence also highlights ferroptosis as a key process in hematopoietic and bone marrow microenvironments, where iron-dependent oxidative damage influences cell survival and function. Therefore, ferroptosis may represent an important mechanistic link between iron overload, oxidative stress, and ineffective erythropoiesis in thalassemia cases, and could serve as a potential therapeutic target [21,23,39].

Herein, we provided several new insights into the relationship between iron metabolism, oxidative stress, and immune cell function in thalassemia. First, our results demonstrate that granulocyte ROS and RAI are significantly impaired in β-thalassemia intermedia while lymphocyte oxidative responses are largely preserved. This finding highlights the potential selective dysfunction of innate immune cells in thalassemia patients, which may have contributed to the increased susceptibility to infections observed in these patients. Second, the study revealed a marked variability and elevation of intracellular GSH levels in BTE patients, suggesting the activation of compensatory antioxidant mechanisms in response to chronic oxidative stress was associated with ineffective erythropoiesis and iron overload. Third, the integration of flow cytometric immune markers, systemic oxidative stress indicators, and MRI-based tissue iron measurements provides a comprehensive evaluation of the complex interactions between iron overload, oxidative damage, and immune dysregulation in thalassemia patients. The study also has several strengths. It combines multiple complementary approaches, including hematological analysis, biochemical assessment, iron metabolism evaluation, flow cytometric immune cell functional analysis, and oxidative stress biomarker measurement, allowing for a multidimensional assessment of disease-related alterations. Furthermore, the inclusion of several comparison groups, including healthy control, IDA, obesity, TT, BTE, and BTM groups, allowed for a clearer interpretation of disease-specific changes in oxidative and immune parameters.

However, several limitations should be acknowledged. First, the sample size of some of the groups, particularly the IDA, obesity, TT, and BTM groups, was relatively small, which may have limited the statistical power required for detecting subtle differences between groups. Second, the cross-sectional design of the study prevented the assessment of temporal changes in oxidative stress and immune function during disease progression or treatment. Third, although important oxidative stress biomarkers were measured, additional markers such as superoxide dismutase, catalase, or inflammatory cytokines were not included and could provide further insight into the underlying mechanisms of immune dysfunction in thalassemia cases. Future research should therefore focus on larger, multicenter cohorts to confirm the observed alterations in oxidative stress and immune cell function. Longitudinal studies evaluating changes in oxidative and immune parameters during transfusion therapy and iron chelation treatment would also be valuable. In addition, further investigations of molecular pathways linking iron overload to immune cell dysfunction, including inflammatory signaling and mitochondrial oxidative stress pathways, may provide important insights into disease mechanisms. Such studies could ultimately contribute to the development of targeted antioxidant or immunomodulatory therapeutic strategies aimed at improving immune function and reducing oxidative damage in patients with thalassemia.

Taken together, the findings of this study highlight the complex interplay between iron overload, oxidative stress, ferroptosis and immune cell dysfunction in thalassemia patients. Granulocyte oxidative impairment appears to be a prominent feature of immune dysregulation in β-thalassemia cases, whereas lymphocyte responses remained relatively preserved. Furthermore, the observed elevation of GSH levels suggested activation of antioxidant defense pathways that may have partially counteracted the oxidative stress in these patients. Overall, these results provide new insights into the relationship between iron metabolism and immune cell function in thalassemia patients. Improved understanding of these mechanisms could contribute to the development of therapeutic strategies aimed at reducing oxidative stress and improving immune function in patients with thalassemia.

## 5. Conclusions

This study demonstrates significant alterations in hematological parameters, iron metabolism, oxidative stress markers, and immune cell function across different clinical groups, particularly in patients with β-thalassemia. Thalassemia patients exhibited severe anemia, elevated liver enzyme activities, and increased bilirubin levels, reflecting enhanced hemolysis and potential hepatic involvement. T2* MRI analysis showed that hepatic iron accumulation occurs earlier and more frequently than cardiac iron deposition, while severe myocardial iron overload was mainly observed in β-thalassemia major. Flow cytometric analysis revealed impaired granulocyte oxidative burst activity in β-thalassemia intermedia, indicating altered innate immune function, whereas lymphocyte responses remained largely preserved. Increased variability in glutathione levels suggests activation of antioxidant defense mechanisms in response to chronic oxidative stress. These findings provide insight into the interplay between iron overload, oxidative stress, and immune dysfunction in thalassemia cases.

## Figures and Tables

**Table 1:** Demographic and clinical characteristics, treatment status, and iron parameters of non-thalassemia participants and thalassemia patients.

Group	Age	Gender	BTX	SPX	Iron Chelation
	(yr)	(M/F)	(Y/N)	(Y/N)	(Y/N)
**Non-thalassemia**					
Healthy (n = 14)	35.6 ± 8.0	2/12	0/14	0/14	0/14
IDA (n = 2)	35.0 ± 5.7	0/2	0/2	0/2	0/2
Obesity (n = 2)	43.5 ± 20.5	1/1	0/2	0/2	0/2
**Thalassemia**					
TT (n = 2)	49.5 ± 7.8	0/2	0/2	0/2	0/2
BTE (n = 28)	38.9 ± 13.6	13/15	28/0	12/16	None (n = 1), DFO (n = 1), DFP (n = 12), DFX (n = 11), Combination (n = 3)
BTM (n = 6)	31.8 ± 6.8	2/4	6/0	5/1	DFO (n = 1), DFX (n = 1), Combination (n = 4)

Abbreviations: BTE = β-thalassemia HbE, BTM = β-thalassemia major, BTX = blood transfusion, DFP = deferiprone, DFO = desferrioxamine, DFX = deferasirox, F = female, IDA = iron deficiency anemia, M = male, N = no, SPX = splenectomy, TT = thalassemia trait, Y = yes, yr = year.

## Data Availability

The original contributions presented in this study are included in the article/Supplementary Materials. Further inquiries can be directed to the corresponding author.

## Supplementary Materials

The following supporting information can be downloaded at: https://www.mdpi.com/article/10.3390/antiox15040482/s1.

## Abbreviations

The following abbreviations are used in this manuscript:

ABTS	2,2-Azino-*bis*-(3-methylbenzothiazoline-6-sulfonic acid)
ABTS^•+^	ABTS radical cation
ALP	Alkaline phosphatase
ALT	Alanine aminotransferase
ANOVA	Analysis of variance
AST	Aspartate aminotransferase
BHT	Butylated hydroxytoluene
BTE	β-Thalassemia HbE
BTM	β-Thalassemia major
BTX	Blood transfusion
CBC	Complete blood count
CRE	Creatinine
DB	Direct bilirubin
DCF	Dichlorofluorescein
DCFH-DA	2′,7′-Dichlorohydrofluorescein diacetate
DFP	Deferiprone
DFO	Desferrioxamine
DFX	Deferasirox
DTNB	5,5-Dithio-bis (2-nitrobenzoic acid)
F	Female
FO	FerroOrange
FSC	Forward scatter
GSH	Reduced glutathione
Hb	Hemoglobin
HbE	Hemoglobin E
HEPES	Hydroxyethyl piperazineethanesulfonic acid
HO	Heme oxygenase
IDA	Iron deficiency anemia
IQR	Interquartile range
LF	Liperfluo
LPO	Lipid peroxide
Lym	Lymphocyte
M	Male
MCH	Mean corpuscular hemoglobin
MCHC	Mean corpuscular hemoglobin
MCV	Mean corpuscular volume
Mon	Monocyte
ms	Millisecond
n	Number
N	No
NA	Not available
ND	Not done
Neu	Neutrophil
OD	Optical density
PB	Phosphate-buffered
PBS	Phosphate-buffered saline
RAI	Redox-active iron
RDW-CV	Red cell distribution width coefficient of variation
REC	Research Ethics Committee
ROIs	Regions of interest
ROS	Reactive oxygen species
SD	Standard deviation
SI	Serum iron
SPX	Splenectomy
SSC	Side scatter
TB	Total bilirubin
TBA	2-Thiobarbituric acid
TBARS	Thiobarbituric acid-reactive substance
TEAC	Total antioxidant capacity
TI	β-Thalassemia intermedia
TIBC	Total iron-binding capacity
TNB	5-Mercapto-2-nitrobenzoic acid
Trolox	6-Hydroxy-2,5,7,8-tetramethylchroman-2-carboxylic acid
TT	Thalassemia trait
T2*-MRI	Magnetic resonance imaging using T2*
UIBC	Unsaturated iron-binding capacity
U/L	Units per liter
Y	Yes
yr	Year
