# Supplementary material for: Iron Overload-Associated Oxidative Stress and Immune Cell Dysfunction in Thalassemia: Integrative Analysis of Hematological, Biochemical, and Flow Cytometric Biomarkers
Source: Antioxidants (Basel). 2026 Apr 14;15(4):482. doi: 10.3390/antiox15040482 (PMC13112990; doi:10.3390/antiox15040482)
Supplement: Supplementary file 1 [file antioxidants-15-00482-s001.zip › antioxidants-4231280-supplementary.pdf]

Supplementary Information

Table S1. Demographic, treatment, and body iron data of normal (n = 14), iron deficiency anemia (n = 2), dyslipidemia (n = 1), obesity (n = 1), thalassemia trait (n = 2), β-thalassemia HbE (n = 28), and β-thalassemia major (n = 6) subjects.

Subject	Diagnosis	Age (yr)	Gender	BTX	SPX	T2*-MRI (msec)		Iron chelation
						Heart	Liver	
C1	Normal	36	F	N	N	N/A	N/A	N
C2	Normal	42	F	N	N	N/A	N/A	N
C3	Normal	47	F	N	N	N/A	N/A	N
C4	Normal	30	M	N	N	N/A	N/A	N
C5	Normal	32	F	N	N	N/A	N/A	N
C6	Normal	38	F	N	N	N/A	N/A	N
C7	Normal	36	F	N	N	N/A	N/A	N
C8	Normal	52	F	N	N	N/A	N/A	N
C9	Normal	30	F	N	N	N/A	N/A	N
C10	Normal	42	F	N	N	N/A	N/A	N
C11	Normal	23	F	N	N	N/A	N/A	N
C12	Normal	30	F	N	N	N/A	N/A	N
C13	Normal	33	M	N	N	N/A	N/A	N
C14	Normal	27	F	N	N	N/A	N/A	N
	**Mean ± SD**	**35.6 ± 8.0**	**2M, 12F**	**N**	**N**	**N/A**	**N/A**	**N**
C15	IDA	39	F	N	N	N/A	N/A	N
C16	IDA	31	F	N	N	N/A	N/A	N
	**Mean ± SD**	**35.0 ± 5.7**	**2F**	**N**	**N**	**N/A**	**NA**	**N**
C17	Obesity	58	F	N	N	N/A	N/A	N
C18	Obesity	29	M	N	N	N/A	N/A	N
	**Mean ± SD**	**43.5 ± 20.5**	**1M, 1F**	**N**	**N**	**N/A**	**NA**	**N**
TT1	Thalassemia trait	55	F	N	N	N/A	N/A	N
TT2	Thalassemia trait	44	F	N	N	N/A	N/A	N
	**Mean ± SD**	**49.5 ± 7.8**	**2F**	**N**	**N**	**N/A**	**N/A**	**N**
BTE1	β-Thalassemia HbE	43	M	Y	N	47.6	11.4	DFP
BTE2	β-Thalassemia HbE	39	M	Y	Y	33.8	15.7	DFX
BTE3	β-Thalassemia HbE	19	F	Y	N	38.0	15.2	DFP
BTE4	β-Thalassemia HbE	68	M	Y	N	44.2	10.2	DFP
BTE5	β-Thalassemia HbE	68	F	Y	N	40.7	16.6	Combination
BTE6	β-Thalassemia HbE	32	M	Y	N	42.6	16.5	DFP

BTE7	β-Thalassemia HbE	37	F	Y	Y	26.6	17.5	Combination
BTE8	β-Thalassemia HbE	31	M	Y	N	39.1	18.4	DFP
BTE9	β-Thalassemia HbE	35	M	Y	Y	NA	NA	DFX
BTE10	β-Thalassemia HbE	49	F	Y	N	48.4	12.2	DFX
BTE11	β-Thalassemia HbE	58	F	Y	N	31.0	12.6	DFX
BTE12	β-Thalassemia HbE	57	F	Y	N	40.8	7.4	DFX
BTE13	β-Thalassemia HbE	43	F	Y	Y	45.7	11.1	None
BTE14	β-Thalassemia HbE	36	F	Y	Y	41.0	1.1	DFX
BTE15	β-Thalassemia HbE	32	M	Y	N	42.8	14.1	DFX
BTE16	β-Thalassemia HbE	41	F	Y	N	NA	NA	DFX
BTE17	β-Thalassemia HbE	20	F	Y	Y	40.8	8.4	DFP
BTE18	β-Thalassemia HbE	40	M	Y	Y	38.3	3.7	DFP
BTE19	β-Thalassemia HbE	31	F	Y	N	40.3	11.2	DFP
BTE20	β-Thalassemia HbE	59	F	Y	N	48.0	6.9	DFP
BTE21	β-Thalassemia HbE	38	M	Y	Y	43.1	13.7	DFP
BTE22	β-Thalassemia HbE	50	F	Y	N	46.7	13.0	DFX
BTE23	β-Thalassemia HbE	34	M	Y	Y	38.4	11.1	DFX
BTE24	β-Thalassemia HbE	23	M	Y	Y	44.5	15.3	Combination
BTE25	β-Thalassemia HbE	31	F	Y	N	36.8	24.9	DFO
BTE26	β-Thalassemia HbE	31	M	Y	Y	42.1	10.3	DFP
BTE27	β-Thalassemia HbE	25	F	Y	Y	28.1	16.7	DFX
BTE28	β-Thalassemia HbE	20	M	Y	N	35.4	15.9	DFP
	**Mean ± SD**	**38.9 ± 13.6**	**13M, 15F**	**28Y**	**12Y, 16N**	**40.18 ± 5.73**	**12.73 ± 4.91**	**1None, 1DFO, 12DFP, 11DFX, 3 Combination**
BTM1	β-Thalassemia major	32	F	Y	N	28.2	7.4	Combination
BTM2	β-Thalassemia major	40	F	Y	Y	20.2	14.5	Combination
BTM3	β-Thalassemia major	28	M	Y	Y	NA	NA	DFO
BTM4	β-Thalassemia major	40	F	Y	Y	17.6	12.9	DFX
BTM5	β-Thalassemia major	24	F	Y	Y	8.0	19.8	Combination
BTM6	β-Thalassemia major	27	M	Y	Y	25.8	7.7	Combination
	**Mean ± SD**	**31.8 ± 6.8**	**2M, 4F**	**6Y**	**5Y, 1N**	**19.96 ± 7.92**	**19.96 ± 5.16**	**1DFO, 1DFX, 4 Combination**

Abbreviation: BTE = β-Thalassemia hemoglobin E, BTM = β-Thalassemia major, BTX = blood transfusion, DFP = deferiprone, DFO = desferrioxamine, DFX = deferasirox, HbE = hemoglobin E (HbE), F = female, IC = iron chelation, IDA = iron deficiency anemia, M = male, N = no, N/A = not available, SD = standard deviation, SPX = splenectomy, TT = thalassemia trait, Y = yes, yr = year.

Table S2. Hematological analysis of blood from normal (n = 14), iron deficiency anemia (n = 2), dyslipidemia (n = 1) and obesity (n = 1), thalassemia trait (n = 2), β-thalassemia HbE (n = 28), and β-thalassemia major (n = 6) subjects.

Subject	Hb (g/dL)	Hct (%)	WBC ($x10^3$/mL)	Percent differential WBC Neu	Lym	Mon	PLT ($x10^5$/mL)	MCV (fL)	MCH (pg)	MCHC (g/dL)	RDW-CV (%)
C1	12.8	40.1	5.03	47.5	44.7	5.0	2.44	97.1	31.0	31.9	14.5
C2	12.2	38.6	5.83	50.8	40.7	5.7	3.16	87.3	27.6	31.6	13.2
C3	12.7	38.9	539	55.2	35.6	4.8	3.43	84.9	27.7	32.6	13.2
C4	13.7	41.8	4.19	57.9	33.4	5.3	1.96	84.6	27.7	32.8	13.0
C5	12.9	40.1	8.65	60.3	27.1	5.9	3.28	89.1	28.7	32.2	13.2
C6	12.2	39.9	9.34	56.9	37.0	5.1	.287	82.3	25.2	30.6	15.9
C7	13.9	42.1	5.02	64.9	26.3	4.8	2.84	90.0	29.7	33.0	13.0
C8	12.5	39.6	10.12	30.0	39.2	4.2	3.12	96.8	30.6	31.6	13.2
C9	13.4	40.1	8.53	67.3	26.7	5.6	3.18	92.8	31.0	33.4	12.2
C10	11.3	35.0	8,28	56.9	32.5	8.1	3.06	94.1	30.4	32.3	12.3
C11	12.5	38.7	7.18	56.3	37.2	5.6	3.11	79.6	25.7	32.3	13.2
C12	14.0	41.9	7.16	50.4	42.3	5.4	2.95	82.2	27.5	33.4	13.3
C13	14.0	46.4	6.23	57.5	32.9	5.1	2.48	81.3	24.5	30.2	13.0
C14	13.0	40.5	5.56	54.6	39.0	5.0	2.89	90.6	29.1	32.1	13.2
**Mean ± SD**	**12.9 ± 0.8**	**40.3 ± 2.5**	**6.89 ± 1.84**	**54.8 ± 8.9**	**35.3 ± 5.8**	**5.4 ± 0.9**	**2.91 ± 38.9**	**88.1 ± 5.8**	**28.3 ± 2.1**	**32.1 ± 0.9**	**13.3 ± 0.9**
C15	10.9	35.8	6.52	50.1	37.6	6.9	2.79	75.2	22.9	30.4	16.9
C16	11.6	35.2	8.90	74.9	13.9	8.5	2.98	86.1	28.4	33.0	12.3
**Mean ± SD**	**11.3 ± 0.5**	**35.5 ± 0.4**	**7.71 ± 1.68**	**62.5 ± 17.5**	**25.8 ± 16.88**	**7.7 ± 1.1**	**2.88 ± 1.34**	**80.7 ± 7.7**	**25.7 ± 3.9**	**31.7 ± 1.8**	**14.6 ± 3.3**
C17	13.2	40.5	4.65	57.5	32.9	6.0	1.96	94.2	30.7	32.6	12.2
C18	14.8	44.8	8.38	54.6	39.0	6.0	3.11	88.5	29.2	33.0	12.6
**Mean ± SD**	**14.0 ± 1.1**	**42.7 ± 3.0**	**6.76 ± 2.99**	**54.8 ± 2.4**	**37.1 ± 3.9**	**6.0 ± 0.0**	**2.54 ± 8.1**	**91.4 ± 4.0**	**30.0 ± 1.1**	**32.8 ± 0.3**	**12.4 ± 0.3**
TT1	11.7	36.9	6.74	48.3	41.2	5.9	2.84	70.3	22.3	31.7	16.0
TT2	11.8	39.5	8.27	53.4	39.3	6.4	3.02	68.7	20.5	29.9	17.0
**Mean ± SD**	**11.8 ± 0.1**	**38.2 ± 1.8**	**7.50 ± 1.08**	**50.9 ± 3.6**	**40.3 ± 1.3**	**6.2 ± 3.4**	**2.93 ± 1.27**	**69.5 ± 1.1**	**21.4 ± 1.3**	**30.8 ± 1.3**	**16.5 ± 0.7**
BTE1	5.6	17.6	3.85	62.1	28.8	5.2	0.58	70.1	22.3	31.8	NA
BTE2	5.7	19.1	6.89	30.8	53.8	14.4	4.91	67.3	20.1	29.8	31.7
BTE3	8.2	25.9	6.35	55.7	37.2	4.6	2.74	65.4	20.7	31.7	32.4
BTE4	6.5	22.7	3.86	63.5	29.8	6.7	1.80	54.4	15.6	28.6	27.2
BTE5	7.0	22.3	4.99	66.4	23.4	5.6	1.03	65.5	20.6	31.4	NA
BTE6	6.0	19.6	4.15	51.8	43.4	3.6	0.93	69	21.1	30.6	27.5
BTE7	7.3	26.4	3.65	44.7	44.6	8.9	3.91	76.5	21.2	27.7	31.9
BTE8	8.9	28.5	11,13	49.6	42.2	5.6	1.89	67.4	21	31.2	NA
BTE9	7.9	24.9	8.02	40.7	45.4	6.5	4.46	70.9	22.5	31.7	NA

BTE10	6.8	21.9	7.68	52.1	35.5	8.1	2.61	62.8	19.5	31.1	34.7
BTE11	8.3	27.5	9.58	59.9	31.3	5.1	2.42	70	21.1	30.2	25.6
BTE12	8.0	24.9	4,.0	43.7	50.7	3.6	1.70	68.4	22	32.1	27.5
BTE13	6.5	21.6	6.17	53.7	38	8.3	7.15	69.7	21	30.1	28.3
BTE14	6.0	18.8	7.46	37.3	47.1	7.6	5.87	75.2	24	31.9	30.9
BTE15	5.5	17.6	4.72	63.1	27.5	7.1	1.63	66.7	20.8	31.3	29.5
BTE16	7.4	24.9	6.56	71.5	22.7	4.6	1.72	58.5	17.4	29.7	29.6
BTE17	8.1	26.5	7.92	48.5	43.3	7.2	9.70	67.9	20.8	30.6	32.8
BTE18	7.6	23.4	1.43	60.5	26.3	13.2	5.02	77.7	25.2	32.5	23.7
BTE19	9.9	29.5	5.00	52	40.8	3.4	1.39	60.6	20.3	33.6	NA
BTE20	9.2	28.0	4.33	47.4	45.7	4.6	1.39	73.5	24.1	32.9	24.5
BTE21	9.5	30.2	2.55	53.3	26.7	13.3	5.28	79.3	24.9	31.5	25.2
BTE22	6.6	21.5	6.28	58.9	34.4	5.3	1.82	61.4	18.9	30.7	31.1
BTE23	8.9	29.0	3.68	68.4	26.3	5.3	5.61	79.7	24.5	30.7	25.6
BTE24	7.2	23.1	7.52	39.8	44.5	13.9	6.13	73.1	22.8	31.2	27.6
BTE25	6.1	18.9	5.67	45.4	45.3	7.9	2.63	75.6	24.4	32.3	28.6
BTE26	6.7	23.3	5.32	47.1	45.9	3.5	6.29	74.9	21.5	28.8	29.3
BTE27	7.5	25.7	5.71	28.9	57.8	2.2	8.55	68.5	20	29.2	31.2
BTE28	7.9	24.6	4,53	55	38.9	4.6	1.59	84.8	27.2	32.1	15.1
**Mean ± SD**	**7.4 ± 1.2**	**23.8 ± 3.7**	**5.69 ± 2.12**	**51.8 ± 10.8**	**38.5 ± 9.5**	**6.8 ± 3.3**	**3.60 ± 2.48**	**69.8 ± 6.9**	**21.6 ± 2.4**	**31.0 ± 1.4**	**28.3 ± 4.1**
BTM1	5.9	18.0	2.72	50	44.1	5.1	1.12	80.4	26.3	32.8	18.5
BTM2	6.9	24.3	8.45	44.8	44.2	6.7	4.49	56.5	16	28.4	26.6
BTM3	8.6	26.2	11.86	45.9	42	9.6	6.31	78.9	25.9	32.8	23.2
BTM4	8.8	27.6	17.09	40.4	50	5.5	3.80	83.9	26.7	31.9	22.2
BTM5	7.7	23.8	13.38	43	49	5.6	7.29	82.4	26.6	32.4	18.4
BTM6	6.7	20.6	11.98	46.9	38.7	12.6	8.04	79.5	25.9	32.5	23.5
**Mean ± SD**	**7.4 ± 1.1**	**23.4 ± 3.6**	**10.91 ± 4.88**	**45.2 ± 3.3**	**44.7 ± 4.2**	**7.5 ± 3.0**	**5.18 ± 2.56**	**76.9 ± 10.2**	**24.6 ± 4.2**	**31.8 ± 1.7**	**22.1 ± 3.2**

Abbreviation: Hb = hemoglobin. Hct = hematocrit, Lym = lymphocyte, MCH = mean corpuscular hemoglobin, MCHC = mean corpuscular hemoglobin concentration, MCV = mean corpuscular volume, Mon = monocyte, Neu = neutrophil, PLT = platelet, RDW-CV = red cell diffraction width-coefficient variation, WBC = white blood cell.

Table S3. Biochemical analysis of serum from normal (n = 14), iron deficiency anemia (n = 2), dyslipidemia (n = 1) and obesity (n = 1), thalassemia trait (n = 2), β-thalassemia HbE (n = 28), and β-thalassemia major (n = 6) subjects.

Subject	AST activity (U/L)	ALT activity (U/L)	ALP activity (U/L)	TB (mg/dL)	DB (mg/dL)	CRE (mg/dL)
C1	14	12	50	0.57	0.23	0.75
C2	22	16	59	0.40	0.20	0.62
C3	13	11	43	0.33	0.23	0.80
C4	20	16	61	0.65	0.21	1.00
C5	10	20	51	0.42	0.31	0.69
C6	16	13	61	0.37	0.18	0.64
C7	14	9	45	0.41	0.22	0.56
C8	19	20	73	0.57	0.17	0.87
C9	15	14	56	0.56	0.25	0.60
C10	14	11	64	1.05	0.43	0.51
C11	18	5	33	0.11	0.06	0.65
C12	12	6	67	0.79	0.31	0.67
C13	25	18	73	0.32	0.15	1.10
C14	12	9	69	0.48	0.21	0.63
**Mean ± SD**	**16.0 ± 4.3**	**12.9 ± 4.8**	**57.5 ± 11.9**	**0.50 ± 0.23**	**0.23 ± 0.09**	**0.72 ± 0.17**
C15	15	4	42	0.37	0.14	0.65
C16	13	10	44	0.54	0.19	0.73
**Mean ± SD**	**14.0 ± 1.4**	**7.0 ± 4.2**	**43.0 ± 1.4**	**0.46 ± 0.12**	**0.17 ± 0.04**	**0.69 ± 0.06**
C17	22	15	54	0.78	0.32	0.80
C18	28	39	57	0.28	0.07	0.80
**Mean ± SD**	**25.0 ± 4.2**	**27.0 ± 17.0**	**55.5 ± 2.1**	**0.53 ± 0.35**	**0.20 ± 0.18**	**0.80 ± 0.00**
TT1	29	19	N/A	N/A	N/A	0.79
TT2	19	14	N/A	N/A	N/A	0.61
**Mean ± SD**	**24.0 ± 7.1**	**16.5 ± 3.5**	**N/A**	**N/A**	**N/A**	**0.7 ± 0.1**
BTE1	17	5	58	1.73	0.8	1.86
BTE2	60	66	NA	NA	NA	0.55
BTE3	32	43	62	2.12	0.79	0.55
BTE4	46	18	52	2.83	1.08	0.78
BTE5	52	45	101	0.77	0.43	0.71
BTE6	51	37	NA	NA	NA	0.72
BTE7	94	75	112	3.59	1.09	0.39
BTE8	49	31	99	2.69	0.84	0.76
BTE9	19	5	83	1.76	1.36	0.81
BTE10	26	25	86	2.12	0.86	0.49
BTE11	34	18	NA	NA	NA	0.61
BTE12	26	8	46	2.46	1.01	0.65
BTE13	27	12	64	2.53	0.87	0.82
BTE14	16	6	86	2.51	0.67	0.46
BTE15	27	23	113	4.25	0.38	0.71
BTE16	51	25	78	4.19	0.49	0.46
BTE17	29	47	80	1.56	0.53	0.45
BTE18	15	12	83	3.43	0.42	0.48
BTE19	25	20	42	1.48	0.49	0.35
BTE20	21	21	NA	NA	NA	0.48
BTE21	26	23	100	1.55	0.53	0.48
BTE22	54	39	NA	NA	NA	0.33
BTE23	18	28	77	3.3	1.05	0.47
BTE24	26	15	133	1.87	0.42	0.52

BTE25	57	51	43	2.24	0.38	0.52
BTE26	28	28	94	1.49	0.43	0.63
BTE27	102	113	78	1.96	0.65	0.47
BTE28	13	13	NA	NA	NA	NA
**Mean ± SD**	**37.2 ± 22.3**	**30.4 ± 24.0**	**80.4 ± 24.1**	**2.38 ± 0.91**	**0.71 ± 0.28**	**0.61 ± 0.29**
BTM1	31	28	79	1.29	0.43	0.45
BTM2	56	28	NA	NA	NA	0.49
BTM3	33	18	79	2.85	0.68	0.57
BTM4	43	45	NA	NA	NA	0.46
BTM5	30	30	104	1.1	0.09	0.4
BTM6	51	42	NA	NA	NA	NA
**Mean ± SD**	**40.7 ± 11.1**	**31.8 ± 10.0**	**87.3 ± 14.4**	**1.75 ± 0.96**	**0.40 ± 0.30**	**0.47 ± 0.06**

Abbreviation: ALP = alkaline phosphatase, ALT = alanine aminotransferase, AST = aspartate aminotransferase, CRE = creatinine, DB = direct bilirubin, TB = total bilirubin, U/L = unit per liter.
